# Towards a Molecular Classification of Sinonasal Carcinomas: Clinical Implications and Opportunities

**DOI:** 10.3390/cancers14061463

**Published:** 2022-03-12

**Authors:** Cecilia Taverna, Abbas Agaimy, Alessandro Franchi

**Affiliations:** 1 Department of Translational Research and of New Technologies in Medicine and Surgery, University of Pisa, 56124 Pisa, Italy; cecitave88@libero.it; 2 Institute of Pathology, Comprehensive Cancer Center (CCC) Erlangen-EMN, 91054 Erlangen, Germany; abbas.agaimy@uk-erlangen.de

**Keywords:** sinonasal carcinomas, pathology, tumor classification, molecular subtyping

## Abstract

**Simple Summary:**

In recent years, there have been several molecular and immunohistochemical additions to the pathologic diagnosis of sinonasal malignancies that could facilitate the identification of clinically relevant groups of sinonasal malignancies. Molecular profiling is progressively integrated in the histopathologic classification of sinonasal carcinomas, and it is likely to influence the management of these tumors in the near future. In this article we review the recent literature on molecular analysis and/or subtyping of sinonasal carcinomas and we discuss the possible clinical implications of a classification of sinonasal tumors based on their molecular features.

**Abstract:**

Sinonasal carcinomas are a heterogeneous group of rare tumors, often with high-grade and/or undifferentiated morphology and aggressive clinical course. In recent years, with increasing molecular testing, unique sinonasal tumor subsets have been identified based on specific genetic alterations, including protein expression, chromosomal translocations, specific gene mutations, or infection by oncogenic viruses. These include, among others, the identification of a subset of sinonasal carcinomas associated with HPV infection, the identification of a subset of squamous cell carcinomas with *EGFR* alterations, and of rare variants with chromosomal translocations (*DEK::AFF2, ETV6::NTRK* and others). The group of sinonasal adenocarcinomas remains very heterogeneous at the molecular level, but some recurrent and potentially targetable genetic alterations have been identified. Finally, poorly differentiated and undifferentiated sinonasal carcinomas have undergone a significant refinement of their subtyping, with the identification of several new novel molecular subgroups, such as NUT carcinoma, *IDH* mutated sinonasal undifferentiated carcinoma and SWI/SNF deficient sinonasal malignancies. Thus, molecular profiling is progressively integrated in the histopathologic classification of sinonasal carcinomas, and it is likely to influence the management of these tumors in the near future. In this review, we summarize the recent developments in the molecular characterization of sinonasal carcinomas and we discuss how these findings are likely to contribute to the classification of this group of rare tumors, with a focus on the potential new opportunities for treatment.

## 1. Introduction

Sinonasal carcinomas are rare and aggressive tumors, with an incidence of less than 1 case per 100,000 population annually, representing 3% to 5% of all head and neck cancers [1,2,3]. They occur predominately in adult male patients and present with nonspecific symptoms that are often indistinguishable from inflammatory diseases. Thus, the diagnosis is often delayed until the tumor is in advanced stage, with frequent involvement of the orbit or the skull base, with limited treatment options. 

Even though the sinonasal tract is a relatively small but complex anatomic region, it may give rise to a wide variety of histologically distinct solid tumors, both epithelial and non-epithelial, and often with high-grade and/or undifferentiated morphology. In recent years, with increasing molecular testing, unique sinonasal tumor subsets have been identified based on specific genetic alterations, including protein expression, chromosomal translocations, infection by oncogenic viruses or specific gene mutations. The 4th Edition of the World Health Organization (WHO) Classification of sinonasal tumors includes the following major groups of epithelial malignancies: keratinizing and non-keratinizing squamous cell carcinoma, spindle cell squamous cell carcinoma, lymphoepithelial carcinoma, sinonasal undifferentiated carcinoma (SNUC), NUT carcinoma, neuroendocrine carcinoma, adenocarcinoma (including intestinal and non-intestinal subtypes), and teratocarcinosarcoma [4]. However, some of these tumor categories, like for example non-intestinal type adenocarcinomas and SNUC, are not yet fully characterized and are currently diagnoses of exclusion, resulting in a heterogeneous collection of tumors referred to by descriptive terminologies of little clinical significance.

Tumor classifications respond to the need for standardization in reporting and tailored therapies and must be based on evidence and validated to be useful in clinical practice. Clinically relevant tumor entities are identified through criteria that must be consistently applied by pathologists in routine practice to identify diagnostic categories that are meaningful for prognostication and treatment. 

Analogously to other anatomic sites, the classification of sinonasal tumors is based on their histologic features, with the broad assumption that the phenotype of neoplastic cells and their lines of differentiation defined with reference to a normal counterpart, often with the help of ancillary techniques such as immunohistochemistry and molecular methods, identify categories that share common pathogenic processes and clinical behavior. However, morphologically similar or even identical tumors can have widely variable clinical course and heterogeneous response to the same treatment, indicating that distinct molecular events driving each cancer type are associated with therapy outcomes.

Generally, tumors present distinct molecular events that drive transformed cell behavior whose identification could ideally help to guide treatment decisions in a personalized way. The categorization of molecular subgroups of cancers has already been translated into clinical practice in some more common tumor types, like for example breast carcinoma patients, whose treatment is currently based on the assessment of predictive markers like proliferative activity, ER, PR and HER2. Similar attempts have been made to classify colorectal and endometrial cancer according to prognostic and predictive molecular markers [5], and molecular subtypes of gliomas have been incorporated in the 2016 WHO central nervous system tumor classification [6]. 

In recent years there have been several molecular and immunohistochemical additions to the pathologic diagnosis of sinonasal malignancies that could facilitate the identification of clinically relevant groups of sinonasal malignancies. However, it should be considered that this approach may be difficult to apply to rare tumors like sinonasal carcinomas, where the results could be the creation of small tumor categories whose clinical significance could be difficult to verify due to lack of statistical power. In this article we review the recent literature on molecular analysis and/or subtyping of sinonasal carcinomas and we discuss the possible clinical implications of a classification of sinonasal tumors based on their molecular features.

## 2. Squamous Cell Carcinoma

Sinonasal squamous cell carcinoma (SNSCC) is the most common subtype of sinonasal malignancy, accounting for 60% to 75% of sinonasal tumors [7,8]. As in other head and neck subsites, SNSCC is divided into keratinizing (KSNSCC) and non-keratinizing (NKSNSCC) variants. Other less frequent subtypes include spindle cell (sarcomatoid), verrucous, basaloid, lymphoepithelial, and adenosquamous carcinoma. Notably, SCC can be synchronous or metachronous to sinonasal papilloma, most frequently the inverted subtype.

Etiology and risk factors are similar in KSNSCC and NKSNSCC, being both correlated to cigarette smoking, with a less strong correlation than in other head and neck subsites, and dust exposure, especially wood and leather [9], even if with a lower proportion of cases than intestinal-type adenocarcinoma. A significant proportion of SNSCC, mainly in the NK category, is associated with high-risk HPV infection, even if the relationship between HPV infection, carcinoma development and its biological significance are still unclear [10]. The most frequently involved sinonasal subsites are the maxillary sinuses, followed by the nasal cavity and ethmoid sinus, with only occasional cases arising in sphenoid and frontal sinuses. As for the vast majority of sinonasal malignancies, signs and symptoms are nonspecific, including epistaxis, nasal obstruction and rhinorrhea, leading to late diagnosis of the disease. 

KSNSCC is composed of nests, cords and island of polygonal epithelial cells interspersed in a desmoplastic stroma, which display abundant eosinophilic cytoplasm, often pleomorphic nuclei and various degrees of intracellular and extracellular keratinization according to the degree of differentiation [4]. NKSNSCC consists of nests and ribbons of atypical squamous cells with high nuclear to cytoplasm ratio, often showing a peripheral palisading, with no intervening desmoplastic stroma. Keratinization is poor or absent, while mitotic figures and necrosis are frequently observed. The tumor often shows a well-delineated pushing invasion front with occasional papillary-like appearance that might be mistaken for a noninvasive lesion. Although infrequently necessary for diagnosis, the immunohistochemistry of KSNSCC shows positivity for cytokeratins of various molecular weights, including cytokeratin 5/6, as well as for P40 and P63 [4]. 

The genomic landscape of SNSCC has been a topic of several studies in the last few years, with interesting results. In general, SNSCC showed several alterations of chromosomal regions with copy number changes similar to those present in head and neck SCC [11]. *TP53* is the most widely aberrant gene in cancer and one of the most studied genes in cancer molecular landscape, together with the expression of its encoded protein, p53. *TP53* has a tumor suppression role, it is mapped on chromosome 17p13 and encodes for p53 protein, which is involved in cell cycle regulation, inhibition of DNA synthesis, and DNA repair and apoptosis [12,13,14]. SNSCC, especially the keratinizing subtype, shows mutation in *TP53* with high frequency, ranging from 33.3% to 100% [15]. Some studies showed a correlation between *TP53* mutations and wood dust exposure and a worse overall survival [15]. Wang and colleagues in a recent meta-analysis tried to correlate the expression of the product of *TP53* to clinico-pathological features in patients with SNSCC [16]. They found that p53 expression is not correlated to histological subtypes, while there is a correlation with tumor differentiation, with poorly differentiated neoplasms having a higher (aberrant) expression of p53 than well differentiated ones, suggesting that p53 may play a role in the progression of SNSCC [16]. 

*EGFR* (epidermal growth factor receptor) gene is a widely studied gene in cancer, and in the last few years it has been used as a treatment target with positive results in head and neck SCC [17,18]. De novo SNSCC presents frequent alterations in *EGFR* including amplifications, copy number gains or activating mutations. *EGFR* gene copy number gains and protein overexpression have been detected in approximately 40% of the cases of SNSCC [19], while *EGFR* activating mutations were found in a lower proportion of cases, ranging between 6 and 15%, and were mainly localized in exons 20 and 19 [20]. However, the prevalence of *EGFR* mutations in SNSCC arising in papillomas is significantly higher (see below for discussion). In addition, *EGFR* copy number increase and protein overexpression were mutually exclusive with *ERBB2* copy number increase, that were observed in another 20% of cases [19]. Similarly, *EGFR* mutations, *EGFR* copy number gains and presence of HR-HPV are essentially mutually exclusive in SNSCC [21]. However, analyses of the correlations between *EGFR* gene status and clinical parameters and survival data have generated controversial results, possibly due to the small number of cases included in each study and the different methods used (immunohistochemistry for protein expression vs. molecular analysis of gene status). Takahashi and coworkers identified *EGFR* as the only predictor of clinical outcome in a study of several potential prognostic markers [22]. Similarly, in a series of 85 SNSCCs studied by Nishikawa et al., patients with *EGFR*-mutated tumors had a worse overall survival than those with *EGFR* wild-type tumors in multivariate analysis [23]. In contrast, other studies failed to identify any clear correlation between *EGFR* status and survival [19,24,25]. A recent study confirmed that *EGFR* copy number gain and protein overexpression can be frequently detected in SNKSCC (30% of the cases), but still no correlation was found with several clinical parameters [26]. Interestingly, in this series of SNSCCs, *EGFR* copy number gain (CNG) and HPV infection were mutually exclusive, and the HPV+/*EGFR* CNG- group had significantly better prognosis than the HPV-*/EGFR* CNG+ group [26]. These results were confirmed in a larger series of 146 SNSCC patients, where the HPV-negative/EGFR-mutant group, the HPV-negative/*EGFR* CNG-positive group, and the triple-negative group had significantly worse prognoses than the HPV-positive group [21]. Thus, molecular subclassification of SNSCCs according to *EGFR* and HPV status may help to select patients for the appropriate treatments and may be a good predictor of prognosis. Moreover, subclassification of SNSCC according to a combination of markers is more likely to be successful in the identification of clinically relevant groups.

While human papillomavirus (HPV) has a well-established role in the development of oropharyngeal carcinoma and HPV-related oropharyngeal carcinoma is considered a specific tumor entity with a distinct TNM classification, the effective oncogenic role and clinical significance of papillomaviruses in sinonasal tract carcinogenesis is still controversial. The percentage of sinonasal carcinomas expressing p16 and/or HPV ranges from 12.5 to 25% [27,28,29], while some studies found transcriptionally active high-risk HPV in a proportion of SNSCC ranging from 11.4 to 31.1% [10,21,30,31,32,33,34]. In agreement with oropharyngeal HPV related SCC the most common HPV genotype found in sinonasal carcinomas is HPV 16, followed by HPV-18, 31 and 33 [32,35].

**Figure 1:**
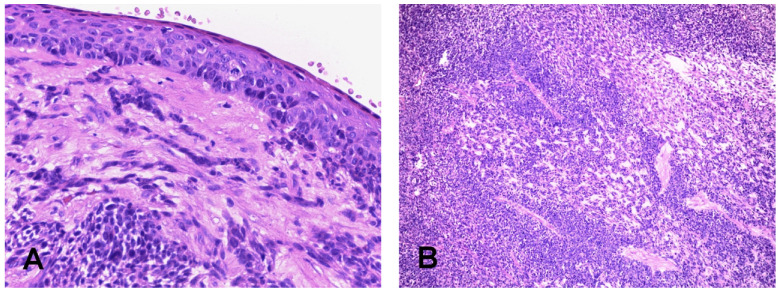
HPV related multiphenotypic carcinoma. The surface epithelium shows severe atypia ((**A**) Hematoxylin and eosin, 20× Objective original magnification). In this example, tumor cells presented a solid growth pattern with large lobules composed of spindle cells organized in fascicles intermixed with areas resembling non-keratinizing squamous cell carcinoma (**B**). Prominent dilated “hemangiopericytoma-like” vessels are present in the background (Hematoxylin and eosin, 10× Objective original magnification).

Among HPV related SNSCC histotypes, NKSCC is more common than KSCC, with frequency being 35–50% versus 4–25% [28,31,36,37,38]. Other histotypes reported to be associated to transcriptionally active HR-HPV are basaloid, papillary and adenosquamous carcinomas with prevalence being respectively 46–56.5%, 42–80% and 66–83% [36,37,38]. In addition, a site-specific entity has been recently added to the list of sinonasal HPV-related carcinomas. HPV-related multiphenotypic sinonasal carcinoma is a distinctive carcinoma type that, as the name implies, presents multiple lines of differentiation, including ductal/myoepithelial and squamous. This results in a mixed histologic appearance with areas resembling a salivary gland-type carcinoma, mainly adenoid cystic carcinoma, epithelial-myoepithelial carcinoma and myoepithelial carcinoma, while squamous differentiation is usually limited to the surface epithelium that presents features of high grade squamous dysplasia/carcinoma in situ, and less frequently consists of truly invasive SCC [39] (Figure 1). By definition, this tumor type harbors predominantly non-16/18 high-risk HPV, usually the rare type 33 [39]. 

However, the lack of consistency in the use of detection assays and the differing study populations that have included both malignant and benign lesions like sinonasal papillomas, have generated disparate findings and have precluded a thorough understanding of the role and significance of HPV infection in sinonasal carcinomas [40]. Another source of confusion may be in the use of p16 positive immunostaining as a surrogate marker for detecting HPV infection. 

While diffuse immunoreactivity for p16 is currently considered a reliable marker for the presence of high-risk HPV in oropharyngeal SCC, this test has in general showed a low predictive value (60–70%) in SNSCC [10,26,32]. However, when tested as prognostic marker in SNSCC, p16 was associated with improved survival, thus being a promising marker for patient stratification [26,41].

Conflicting data have been reported regarding the clinical significance and the prognostic impact of HPV positivity in sinonasal SCC. Although general agreement is still lacking, in most studies HPV positive SNSCC showed a favorable prognosis, with a trend towards improved overall survival and disease-free survival in comparison with HPV negative SNSCC [26,30,31,32,33,37,41]. 

A proportion of sinonasal carcinomas are synchronous or metachronous with sinonasal papilloma (SP). Malignant transformation can occur in a percentage of cases between 11% and 19% [42,43] and it is more commonly detected at the time of the first diagnosis of SP (64%) than in recurrent SPs (36%) [43]. The most frequent histotype is KSCC, although NKSCC, mucoepidermoid carcinoma, sinonasal undifferentiated carcinoma and verrucous SCC have been reported. The frequency of malignant transformation depends on the types of SPs, with inverted SPs (ISP) having the higher risk of malignant transformation, (1.9% to 27%), followed by oncocytic SPs (OSP) (4% to 17%) [43]. Exophytic SPs (ESP) have a very low risk of malignant transformation, with less than 10 cases described in literature [44,45].

The molecular background of SNSCC arising from SPs has been the object of a number of studies, indicating that it could represent a separate biological entity. In particular, *EGFR* was found to be mutated in a significant proportion of SNSCC arising from sinonasal papillomas, with the most frequent alteration being gene copy number gain and insertion (mostly exon 20 insertion and deletions and single-nucleotide substitutions in exon 19 and 20) [20,25,34]. Inverted SP is the only subtype that harbors EGFR alterations, with variable frequency in different studies, and the same molecular signature is usually found in the malignant SCC component. Indeed, Udager et al. found that *EGFR* activating mutations are present in 88% of ISPs and 77% of SNSCC associated with ISPs [46] while Sasaki et al. found the same trend of mutation in 88% of SNSCC arising from SPs [20]. These findings suggest that *EGFR* mutation is an early event in ISP pathogenesis and ISPs/ISPs-related SNSCC are a separate molecular family of neoplasms, as no evidence of *EGFR* mutation was found in OSPs and ESPs. 

Hongo and colleagues studied a series of SNSCC including cases arising from ISPs, and correlated the *EGFR* status, both gene copy number gain (CNG) and mutations, as well as the presence of HPV infection with clinical features [21]. They found that HR-HPV was present in a minority of SPs cases (7.5%), while it was absent in SNSCC-IPs related. *EGFR* mutations were found in 21% of SNSCC, the majority being ISPs associated (92.2%) and *EGFR* CNG were seen in 28.1% of the cases. The HPV-negative/*EGFR*-mutant group, the HPV-negative/*EGFR*-CNG positive group and the triple negative group had a worse prognosis than the HPV positive one. Moreover, *EGFR* mutations and HPV infection are mutually exclusive events in the carcinogenesis of SNSCC arising from ISP [47,48].

In summary, *EGFR* exon 20 insertion mutations are frequently detected in SNSCC arising in ISP and very rarely in SNSCC arising de novo [20,25]. This observation indicates that the detection of *EGFR* mutations in SNSCC is strongly supportive of its origin from ISP. Conversely, *EGFR* copy number gains are found in SNSCC not arising in ISP or OSP [21].

*KRAS* is another gene frequently altered in both OSPs and SNSCC arising in OSPs. Udager et al. first reported the presence of *KRAS* mutations in all cases of OSPs and OSPs-related SNSCC, with 4 out of 5 matched OSP and associated sinonasal SCC having concordant *KRAS* genotype [49]. In a recent study, Brown and colleagues confirmed the presence of frequent *KRAS* mutations in OSP and associated squamous cell carcinoma [50]. As for EGFR, the presence of *KRAS* mutations in a subset of SPs and related SCC supports the idea that they are a separate entity with a distinct biological history and mechanism of carcinogenesis. *KRAS* can also be a promising target for new and personalized treatments, even though FDA-approved drugs are not available at the moment [51] and it is an important molecular feature to evaluate as the presence of *KRAS* mutation seems to predict resistance to anti-*EGFR*-based therapy [51]. Brown and colleagues also studied *TP53* and *CDKN2A* status in SNSCC arising in SP and found that almost all of them (96.6%) harbor TP53 mutations or inactivating mutations with loss of heterozygosis or two copy loss of *CDKN2A* [50]. Interestingly, these molecular alterations are absent in sinonasal papillomas, suggesting that these are key molecular events occurring in the early phases of malignant transformation of SP. They also showed that frequent alterations of *TP53* and *CDKN2A* are related to tobacco exposure, as it happens in SCC of other head and neck subsites [50]. Other minor molecular alterations found in this study were *TERT* copy number gains (27.6%) without *TERT* promoter mutations, *NFE2L2*, *SOX2*, *CCND1*, *MYC*, *FGFR1*, and *EGFR* copy number gains, which are commonly found also in SCC of the aerodigestive tract [50].

The oncogenic role of HPV infection in SNSCC arising from SP is another controversial topic, and the hypothesis the HR-HPV is not associated with SNSCC has been confirmed by several studies in the last few years. While HR-HPV can be present in a small subset of ISPs, SNSCC arising in ISP does not harbor HR-HPV [21,52]. Other studies show that, in contrast to the pathogenic mechanisms observed in the oropharynx and in female genital tract, the most common genotypes of HPV related to SNSCC arising from ISP are within the low-risk group (LR-HPV). Indeed, Udager and colleagues found that while LR-HPV infection is frequently found in ISPs-associated SNSCC, whereas HR-HPV infection is more typical of de novo SNSCC [47]. In agreement, Mehrad et al. found that a subset of ISPs carrying LR-HPV and lacking *EGFR* mutation have a higher risk of malignant transformation [48]. Interestingly, viral integration of LR-HPV genotypes has been found only in carcinoma but not in the precursor IP [53]. Thus, a small subset of IPs may progress to carcinoma with the contribute of LR-HPV, through a mechanism that does not involve degradation of p53 and p16/cyclin D1 dysregulation [53].

**Figure 2:**
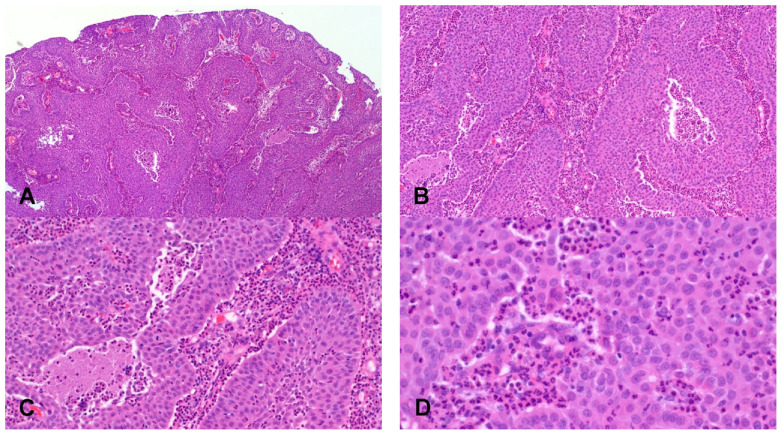
Sinonasal DEK-AFF2 fusion associated squamous carcinoma. The tumor shows an inverted growth pattern ((**A**), Hematoxylin and eosin, 5× Objective original magnification) and consists of nests and interconnecting trabeculae with no evidence of keratinization (**B**), (Hematoxylin and eosin, 10× Objective original magnification). Neutrophilic infiltrates are present both in the stroma and within the tumor ((**C**), Hematoxylin and eosin, 20× Objective original magnification). Tumor cells present a bland uniform appearance with no overt cytologic atypia ((**D**), Hematoxylin and eosin, 40× Objective original magnification).

A recently described molecularly distinct variant of SNSCC is the *DEK::AFF2* fusion-associated carcinoma [54,55,56]. These tumors are not limited to the sinonasal region but may also originate in the skull base and temporal bone. They occur over a wide age range (18–79 years, mean 69 years) with a predominance in females. Histologically, most tumors presented a mixture of exophytic and inverted papillary growth patterns, often with peripheral palisading (Figure 2). Neoplastic cells show a relatively uniform basaloid to polygonal/transitional appearance. Although the majority of these carcinomas have features of NKSCC, foci of keratinization can be present. Gland formation with mucin accumulation has been observed in one case [56]. Another recurrent feature is the presence of a prominent infiltrate of neutrophils or stromal lymphocytes. Mitotic activity is generally low, and foci of necrosis can be present.

The immunoprofile of *DEK::AFF2* carcinomas is in accordance with a squamous phenotype, being diffusely p63 and p40 positive. Both HPV and EBV testing has been negative. The *DEK::AFF2* fusion has been demonstrated by targeted RNA sequencing [56] and confirmed by RT-PCR and by FISH with a *DEK* break-apart probe [55]. Notably, there has been no evidence of *EGFR* or *KRAS* mutations [55,56], indicating that these carcinomas are not related to SP. Although the number of cases reported so far is small, these carcinomas generally tend to have an aggressive behavior, with occurrence of cervical lymph node metastases and distant metastases [55,56]. Although one patient presented an excellent response to multimodality treatments including immune checkpoint inhibitors, this result has not been confirmed in other patients [56].

A further example of a translocation associated SNSCC has been reported in a 66-year-old man. The tumor originated in the sphenoid sinus and histologically showed an undifferentiated morphology being composed by sheets of epithelioid cells with a brisk inflammatory infiltrate [57]. Although there was no histologic evidence of squamous differentiation, there was diffuse immunopositivity for p63 and p40, supporting the diagnosis of NKSCC. Targeted RNA sequencing revealed a novel *ETV6::TNFRSF8* fusion [57]. In situ hybridization for Epstein-Barr virus-encoded small RNAs (EBER) was negative, as was p16. 

In summary, the category of SNSCC presents distinct molecular subgroups (Table 1) with potential clinical importance, which have been progressively refined through the identification of new molecular findings, including novel translocations. Although these translocation-related sinonasal carcinomas often present a histologic profile that falls within the spectrum of SCC, further studies are needed to determine whether they represent SCC variants or entirely separate entities.

Data regarding the clinical importance of molecular characterization of SNSCC are beginning to emerge. Although the clinical evaluation of the efficacy of immune checkpoint inhibitors in sinonasal cancer is still in its early phases, preliminary data indicate that response to treatment may be associated not only to immune marker status (i.e., PDL1 expression, tumor infiltrating lymphocytes and tumor microenvironment subtype) but also to tumor genotypes [58]. Specifically, SNSCCs with *EGFR* mutation showed an unfavorable response to treatment with immune checkpoint inhibitors, whereas in patients with *EGFR* wild type SNSCC this treatment significantly improved the overall survival [58]. Thus, it is likely that in the near future molecular characterization of SNSCC, will become part of the routine assessment of these tumors, in order to support the choice of the best treatment option.

## 3. Adenocarcinomas

Adenocarcinomas are the second most common malignancy of the sinonasal region. They are thought to originate from the surface epithelium or from the seromucous glands of the sinonasal mucosa. Excluding salivary-type tumors, that replicate the clinico-pathologic features of tumors of the salivary glands of the oral cavity, they can be categorized into intestinal-type and non-intestinal type.

Intestinal type adenocarcinoma (ITAC) is mainly related to exposures to wood dust, derived particularly from hardwood species, and leather dusts [9]. It affects more commonly male subjects and originates preferentially in the ethmoid sinus. Histologically, ITAC closely resembles adenocarcinomas or adenomas of intestinal origin and consists of a proliferation of dysplastic columnar cells with interspersed goblet cells, forming papillae and glands [59]. Neoplastic cells are positive for markers of intestinal differentiation, including cytokeratin 20, CDX2, MUC2, and villin [4].

According to the architectural and cytologic features, different subtypes can be identified, including papillary, colonic, solid, mucinous including signet ring cell, and mixed types [59]. Kleinsasser and Schroeder defined papillary-tubular cylindrical cell type (including the papillary, colonic, and solid types), alveolar goblet cell type, signet-ring cell type (corresponding to mucinous type), and transitional cell type (corresponding to mixed type) [60]. While well differentiated papillary tumors have a relatively indolent course, poorly differentiated/solid and mucinous subtypes show a significantly worst prognosis [59,60,61]. This may suggest the existence of different molecular mechanisms of tumor progression according to the different histologic subtypes [62,63,64]. 

In general, the genetic alterations found in sinonasal ITAC are only partially similar to those observed in colorectal cancer [65,66]. Comparative genomic hybridization (CGH) and microarray CGH studies have shown that ITACs harbor complex karyotypes with copy number alterations involving all chromosomes. Hotspot gains have been found at 5p, 7, 8q, 12p, and 20q, while losses were detected at 4q, 5q, 8p, 17p, and 18q [64]. Some recurrent copy number alterations appear to be associated with unfavorable prognosis, specifically, gains at 1q22-23, 3q28-29, 6p22, and 13q31-33, and losses at 4p15-16, 4q32-35, and 10q24, were significantly associated with poor outcome [64]. *TP53* is the most frequently mutated gene (40–50%) [15,65,66,67], while mutations of *APC, KRAS* and *BRAF* occur at a low frequency [68,69,70,71,72]. High levels of EGFR expression and gene amplification have been detected in a subset of ITACs [72,73], while overexpression of MET [74], and nuclear beta-catenin expression are frequently present [75,76]. By applying next generation sequencing techniques to screen for gene mutations in ITAC, recurrent somatic sequence variants were identified in *PIK3CA, APC, ATM, KRAS, NF1, LRP1B, BRCA1, ERBB3, CTNNB1, NOTCH2* and *CDKN2A* [77]. These variants mainly affected PI3K, *MAPK/ERK, WNT* and DNA repair signaling pathways, although not in a mutually exclusive manner and without any clear correlation with clinical parameters or etiology [77]. 

Non-intestinal type sinonasal adenocarcinomas (non-ITAC) represent a heterogeneous category including low-grade and high-grade gland-forming malignancies that do not present an intestinal phenotype. They occur over a wide age range, with a mean age at presentation in the sixth decade and involve preferentially the nasal cavities and maxillary sinus. 

Low-grade non-ITAC are well differentiated tumors that consist in most cases of papillae with fibrovascular cores lined by columnar or cuboidal cells, with absent to slight atypia [4]. In other instances, the core of the tumor consists of small tubules and trabeculae arranged back-to-back and lined by a single layer of cuboidal to columnar cells with slightly eosinophilic cytoplasm. Mucin-producing or oncocytic cells can be present as well as occasional basal cells. These tumors present infiltrative borders, but other features of aggressiveness, such as perineural and lymphovascular invasion, brisk mitotic activity and necrosis are absent. They often present a seromucinous phenotype, as indicated by the expression of S100, DOG1 and SOX10 [78,79], and at least a subset is thought to originate from the terminal duct of the sinonasal seromucinous glands [79,80]. Interestingly, low grade non-ITAC may arise in association with benign lesions, such as sinonasal papillomas, respiratory epithelial adenomatoid hamartomas and sinonasal seromucinous hamartoma [81,82,83]. These associations and the overlapping morphologic and immunohistochemical features of low grade non-ITAC and respiratory adenomatoid hamartoma/seromucinous hamartoma suggest that these benign lesions may be the precursors of low grade non-ITAC [83].

A subset of sinonasal low grade ACs belongs to the group of translocation-associated carcinomas. Indeed, *ETV6*-gene rearrangements with *NTRK3* or *RET* have been reported in low grade ACs with distinctive but varied histology. These segregate into two major subgroups sharing the same genotypes: conventional low-grade (mostly tubulopapillary) non-ITAC and salivary type secretory carcinomas. Although this morphology-based separation of tumors sharing same genotype seems unjustified at first glance, the tendency of those tumors recapitulating salivary secretory carcinomas to behave more aggressive and to harbor a higher-grade component might justify their separation. These tumors also share comparable immunophenotype characterized by positivity for cytokeratin 7, DOG1, S100 and SOX10 [84,85,86]. It is important to be aware of this genetic relationship between these tumor types in view of the more aggressive behavior of secretory carcinoma and for the opportunity of undertaking treatments with NTRK inhibitors [87]. 

A further example of sinonasal low-grade non-intestinal-type adenocarcinoma presented a novel a *SYN2::PPARG* gene fusion [88]. Notably, this tumor also presented some peculiar histologic features, including a predominant tubular architecture with foci of morular metaplasia, and aberrant CDX2 expression both within the morular areas and in the tubular component [89]. Other genetic findings in low grade non-ITAC include the presence of missense mutations in *CTNNB1*, the gene encoding beta-catenin, in two examples showing squamoid morular metaplasia associated with CDX2 immunohistochemical positivity [89], the presence of *BRAF* exon 15 (V600E) T>A mutation [72], and the absence of *TP53, EGFR* and *KRAS* mutations [72,90].

A subset of low grade non-ITAC consists of a uniform population of cuboidal to columnar cells with glycogen-rich clear cytoplasm without mucin production. These tumors resemble quite closely metastatic renal carcinoma and have been designated sinonasal renal cell-like adenocarcinoma [91,92,93]. The architecture is typically follicular/glandular and only occasionally papillae or solid areas are identified. Tumor cells are strongly positive for cytokeratin 7 and carbonic anhydrase IX (CAIX), express CD10 but are negative for PAX-8 and RCC, allowing the distinction from metastatic renal cell clear cell carcinoma [93]. Since CAIX is widely expressed through the seromucinous glands of the Schneiderian mucosa being possibly involved in regulating the ion concentration of sinonasal secretion, it is conceivable that renal cell-like adenocarcinoma may arise from these seromucinous glands [93]. 

The group of high-grade non-ITAC is extremely heterogeneous and poorly characterized, and separation of distinctive categories based on reproducible histologic, immunohistochemical and molecular data is needed. Histologically, most tumors show an undifferentiated predominantly solid architecture, with interspersed glands or occasional micropapillae [94]. Neoplastic cells are small to medium-sized and have scant lightly eosinophilic cytoplasm. Other tumors consist of infiltrating islands and glands formed by larger cells, with abundant eosinophilic cytoplasm and somewhat apocrine features, resembling ductal carcinoma of the breast or high-grade salivary duct carcinoma [95]. Less frequently, the tumor consists of oncocytic cells sometimes mixed with mucinous cells and abundant extracellular mucus. These adenocarcinomas may arise in association with oncocytic sinonasal papilloma [94]. 

The boundaries between these high-grade adenocarcinomas and other high grade sinonasal malignancies like teratocarcinosarcoma or SMARCB1 deficient carcinomas are not well defined. Recently, a series of high grade sinonasal adenocarcinomas with loss of SMARCB1 has been reported [96]. These tumors present true glandular differentiation with formation of cribriform structures and tubules with intracellular and/or intraluminal mucin and may also exhibit areas with yolk sac tumor-like morphology, consisting mainly of microcystic and reticular growth patterns in a myxoid stroma [96]. In parallel, the immunohistochemical profile of these adenocarcinomas is characterized by the loss of INI-1 expression, positivity for CK7, CK20 (focal) and CDX2 (focal), as well as positivity for yolk sac markers like glypican-3, alpha fetoprotein and SALL4 [96]. The separation of this new tumor type from the poorly defined category of high grade non-ITACs is particularly relevant in view of the emerging opportunities for targeted treatments in SMARCB1 deficient tumors (see below for discussion).

In summary, although the distinction between ITAC and non-ITAC is of clinical utility, each category remains quite heterogeneous both morphologically and at the molecular level. Even if the current knowledge is not sufficient to allow a molecular grouping of sinonasal adenocarcinomas, there are emerging evidence that some adenocarcinomas present druggable molecular alterations with the potential for improvement in treatment options for patients.

## 4. Undifferentiated Carcinomas

Sinonasal carcinomas often presents with a poorly differentiated/undifferentiated morphology that makes their evaluation challenging for pathologists, including both the distinction from non-epithelial mimics (lymphomas, sarcomas, melanoma, etc.) and the identification of specific histologic subtypes. Since the description of sinonasal undifferentiated carcinoma (SNUC) in 1986 [97], the group of poorly differentiated sinonasal carcinomas underwent a continuous refinement of their subtyping. Although SNUC was initially described as a distinctive morphologic entity, the lack of a specific line of differentiation and a diagnostic definition of exclusion (lacking evidence of squamous or glandular differentiation by histology and immunophenotyping), led to the inclusion of several poorly differentiated carcinomas in this category. Thus, SNUC has progressively become a morphologic pattern synonymous with undifferentiated carcinoma NOS, instead of a distinctive entity [98].

However, in recent years the identification of new immunohistochemical markers and the advances in molecular profiling of sinonasal neoplasms have improved our capacity to separate undifferentiated sinonasal carcinomas into more meaningful diagnostic categories, with an impact on their classification, assessment of prognosis and treatment. To date, a number of sinonasal tumor entities, which previously fell in the spectrum of SNUC can be separated by their genetic and phenotypic findings. These include NUT carcinoma [99], SMARCB1-deficient carcinoma [100], and SMARCA4 deficient carcinoma [101]. In addition, *IDH2* mutations have been detected in SNUC with variable frequency (31–82%), using immunohistochemistry and/or gene sequencing [102,103,104,105,106], allowing a more solid definition of SNUC.

Table 2 summarizes the pathological and molecular features of sinonasal poorly differentiated and undifferentiated carcinomas classified according to their molecular characterization. 

Even considering the limit that the data collected so far have been obtained from few studies on relatively small series of patients, some relevant correlations are emerging. Dogan et al. first reported a DNA methylation-based classification of sinonasal undifferentiated carcinomas showing that the category of *IDH2* mutant carcinomas, mainly including SNUC and large cell NEC, formed a distinct cluster segregated from other groups [107]. *IDH2* mutated carcinomas presented other distinctive molecular features including a global methylation phenotype and an increase in repressive trimethylation of *H3K27* in comparison to *IDH2* wild-type tumors [107]. Notably, this group of high-grade carcinomas presented a better disease-free survival and lower propensity for lung metastasis than SMARCB1 deficient sinonasal carcinomas. Similarly, Riobello et al. analyzed a series of 125 poorly differentiated and undifferentiated carcinomas for IDH mutations and found that, irrespective of the histologic subtype, disease-specific survival was more favorable in IDH2-mutant versus wild-type cases [106]. In line with these findings, Glöss et al. recently reported a better disease-free survival for IDH2-mutant sinonasal carcinomas in comparison with SMARCB1 deficient carcinomas and IDH2 wild type carcinomas in a large series of sinonasal/skull base tumors [108]. Thus, these data indicate that IDH-mutant sinonasal carcinomas may represent a distinct tumor entity with less aggressive clinical behavior possibly susceptible to treatment with IDH-guided therapies. 

A second group of molecularly defined undifferentiated sinonasal carcinomas presents loss of one SWI/SNF (SWItch/Sucrose Nonfermentable) chromatin remodeling complex subunit, usually either SMARCB1 or SMARCA4. A few cases also present additional loss of the SMARCA2 subunit. SWI/SNF inactivation and *IDH2* mutations seem mutually exclusive, suggesting that these are true driver genetic events in the harboring tumors. 

**Figure 3:**
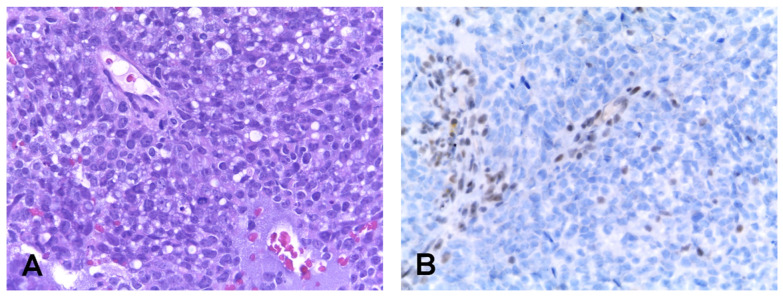
SMARCB1 deficient carcinoma. The tumor consists of a relatively uniform population of undifferentiated basaloid cells organized in solid sheets (**A**). Immunohistochemistry shows complete loss of INI1 in neoplastic cells, while nuclear positivity is retained in stromal cells (**B**).

SMARCB1 (INI1) deficient carcinoma is a rare tumor that develops over a wide age range (median 52 years) with a slight predilection for male patients [100]. Most patients present with a locally advanced disease and, although some patients present long survival with multimodal treatments, the prognosis is generally poor. The histologic and immunophenotypic features of SMARCB1 deficient carcinoma are heterogenous. The most common histologic presentation is that of a relatively uniform population of undifferentiated basaloid cells with round nuclei containing dispersed chromatin and variably prominent nucleoli, organized in solid sheets and nests which are surrounded by desmoplastic stroma (Figure 3). A second population of singly dispersed rhabdoid/plasmacytoid cells, with abundant eosinophilic cytoplasm and eccentrically placed nucleus, can often be identified through a careful search. In approximately one third of the cases the tumor consists predominantly of these rhabdoid/plasmacytoid cells. There is no evidence of squamous differentiation and no signs of dysplasia of the surface epithelium, although the tumor growth may occasionally give rise to superficial spread with replacement the surface epithelium and the glands that may simulate the inverted growth of sinonasal papilloma.

By definition, neoplastic cells present loss of nuclear expression of SMARCB1 (INI1), while the immunostaining is retained in stromal fibroblasts, endothelial cells and inflammatory infiltrate. In addition, SMARCB1 deficient carcinomas are consistently positive for cytokeratins, including variable positivity for CK5 and CK7, while p63 is expressed in approximately half of the cases [99]. Focal positivity for neuroendocrine markers, including CD56, synaptophysin, and chromogranin A has been noted [100]. In addition, p16 may be occasionally positive, but high-risk HPV testing has always been negative [100].

As pointed in the adenocarcinoma section, a subset of SMARCB1 deficient carcinomas presents a predominant oncocytoid/plasmacytoid cytology together with true glandular differentiation consisting of the formation of cribriform structures and tubules with intracellular and/or intraluminal mucin. These rare cases have been designated as “SMARCB1 (INI-1)-deficient adenocarcinoma” [96]. Interestingly, foci with yolk sac tumor-like morphology, consisting mainly of microcystic and reticular growth patterns in a myxoid stroma, may also be present. The immunohistochemical profile of these adenocarcinomas is more complex, and, besides the loss of INI-1 expression, includes positivity for CK7, CK20 (focal) and CDX2 (focal), as well as for yolk sac markers including glypican-3, alpha fetoprotein and SALL4 [96].

**Figure 4:**
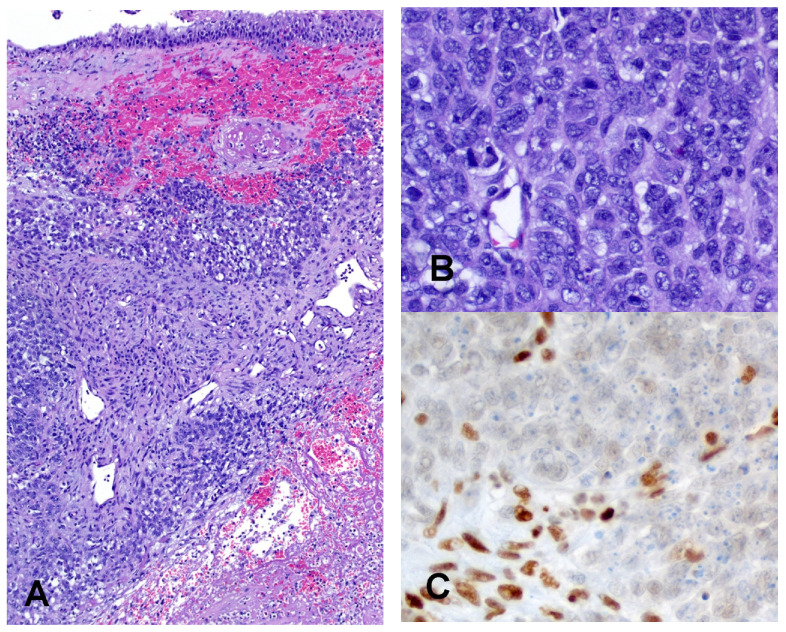
SMARCA4 deficient sinonasal carcinoma. Nests of undifferentiated tumor cells are present in the nasal mucosa (**A**) (Hematoxylin and eosin, 10× Objective original magnification). Tumor cells are large and show vesicular nuclei with multiple nucleoli and moderate amounts of cytoplasm (**B**) (Hematoxylin and eosin, 40× Objective original magnification). Immunohistochemistry shows complete loss of SMARCA4 in neoplastic cells, while nuclear positivity is retained in stromal cells (**C**).

SMARCA4 deficient carcinoma is vanishingly rare in the sinonasal region. Histologically, it is an undifferentiated carcinoma that consists of nests of monomorphic cells that tend to merge in solid sheets with areas of coagulative necrosis [101]. While in the majority of the cases a large cell population predominates, in some cases the tumor cells resemble those of small cell neuroendocrine carcinoma or tend to be more elongated with neuroepithelial-like elements (Figure 4). These tumors show complete loss of SMARCA4 and retained expression of SMARCB1/INI1, while co-loss of SMARCA2 may be present. Immunohistochemically, SMARCA4 carcinomas lack any expression of markers of squamous differentiation, but may show expression of neuroendocrine markers, thus showing a significant phenotypic overlap with neuroendocrine carcinomas, that represent the main differential diagnosis [101].

**Figure 5:**
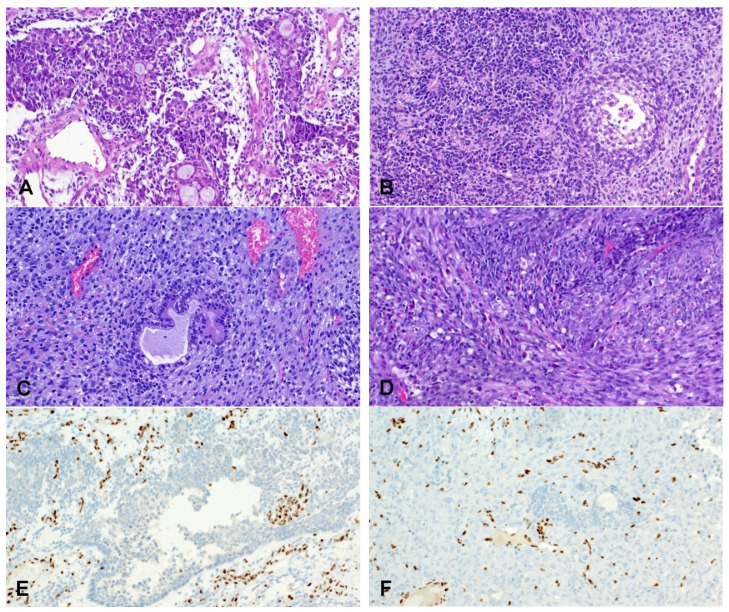
Examples of sinonasal teratocarcinosarcoma showing the triphasic pattern. The epithelial component varies from disordered mucous glands (**A**) to primitive clear cell fetal-type squamous (**B**) or tubular (**C**) structures (Hematoxylin and eosin, 10× Objective original magnification). The stroma varies from primitive neuroectodermal-type (**B**) to lose spindled or primitive non-descript (**C**). Variable rhabdomyoblastic differentiation is frequent (**D**). Global loss of SMARCA4 in the epithelial (**E**) and the primitive and mesenchymal stroma (**F**).

In addition, the loss of SMARCA4 has also been reported in teratocarcinosarcoma, a rare and aggressive sinonasal tumor with multiple lines of differentiation and the presence of teratomatous elements [109] (Figure 5). Interestingly, yolk sac tumor elements have been observed in SMARCB1 deficient carcinomas and adenocarcinomas [96,110,111] and could represent a morphologic link between different entities in the spectrum of SWI/SNF complex deficient malignancies of the sinonasal tract.

NUT carcinoma is a poorly differentiated carcinoma which is defined by a rearrangement of the nuclear protein in testis (*NUTM1*) gene on chromosome 15q14. The most common translocation is the t(15;19) (q13;p13.1), that fuses the *NUT* gene to the *BRD4* gene. Histologically, it is a poorly differentiated carcinoma often presenting evidence of squamous differentiation, which consists of sheets a relatively uniform population of undifferentiated round/polygonal cells [4,112]. Foci of mature keratinized squamous cells may be occasionally seen abruptly juxtaposed to the undifferentiated component [4,112]. Brisk mitotic activity, apoptotic bodies, and areas of necrosis are often recognized. The diagnosis requires the identification of NUTM1 gene rearrangement by FISH or RT-PCR, but diffuse (>50%) immunohistochemical nuclear staining for NUT, usually with a characteristic speckled appearance, is considered sensitive and specific enough to support the diagnosis [4,112].

The standard treatment for undifferentiated sinonasal carcinomas has been so far based on multimodal therapy, including surgery (when feasible), and adjuvant radiation or chemo-radiation. Considering that the frequent locally advanced stage of these aggressive tumors often precludes complete surgical removal, neoadjuvant chemotherapy and adjuvant radiation have shown improved responses, but local relapses are still frequent and overall survival remains poor [113]. Thus, the identification of novel molecular subgroups of undifferentiated sinonasal carcinomas may represent the basis for the translation of personalized cancer medicine into the clinical management of sinonasal cancers with potentially targetable therapeutic options.

In this regard, patients affected by *IDH*-mutated poorly differentiated and undifferentiated sinonasal carcinomas could benefit from specific treatments with mutant IDH inhibitors [114], while SWI/SNF-deficient carcinomas could be targeted by inhibitors of EZH2 [115,116]. Bromodomain inhibitors have been tested for treatment of NUT carcinomas but have shown limited efficacy in vivo [117,118]. These preliminary results emphasize the growing importance of the correct classification of these undifferentiated sinonasal malignancies and support the use of immunohistochemical and/or molecular methods in the routine histopathologic assessment for their clinically relevant characterization. However, it should be noted that, with the only possible exception of NUT carcinoma, genetic grouping of sinonasal undifferentiated and poorly differentiated carcinomas does not match with the currently recognized histopathologic categories. 

## 5. Conclusions

In recent years, there have been several efforts to provide insights into the molecular features of sinonasal carcinomas. These have resulted in the identification of several new tumor entities mainly derived from histologically undifferentiated or poorly defined tumor groups, that likely will be included in the upcoming tumor classification schemes. However, some tumor categories such as sinonasal adenocarcinomas are very heterogeneous at the molecular level. Although some recurrent and potentially targetable genetic alterations have been identified, new clinicopathologic entities with characteristic genetic, histological and immunophenotypic features are yet to be identified. Thus, molecular profiling is progressively integrating the histopathologic classification of sinonasal carcinomas, and it is likely to influence the management of these tumors in the near future.

## Figures and Tables

**Table 1:** Molecular subgroups of sinonasal squamous cell carcinoma.

Molecular Subgroup	Defining Genetic Findings	Histologic Subtype/Features	Other Genetic Findings	Clinical Significance
*EGFR* amplification	Copy number gains	De novo keratinizing SCC (30%)	Absence of *KRAS* mutations	ND
*EGFR* mutated	Mainly *EGFR* exon 20 mutations; rare involvement of exon 19	SCC arising in ISP (90–95%); de novo keratinizing SCC (6–15%)	Absence of *KRAS* mutations; recurrent *TP53* and *CDKN2A* mutations in SCC arising in SP	Worse survival in some studies; potential targeted treatment
*KRAS* mutated	G12V and G12D mutations	SCC arising in OSP (100%)	ND	ND
HR-HPV related (monotypic)	Mainly HPV 16; HPV-18, 31, and 33 rarely detected	Non-keratinizing SCC (50%); Keratinizing SCC (4–25%)	ND	Favorable prognosis
HR-HPV related multiphenotypic carcinoma	Mainly HPV 33, rarely 52, 56 and others	Non-keratinizing SCC (50%); Keratinizing SCC (4–25%)	ND	Favorable prognosis
*DEK::AFF2* translocated	*DEK::AFF2* fusion	Exophytic and endophytic growth; non keratinizing SCC; less frequently keratinizing	Negative for *EGFR* and *KRAS* mutations; absence of HR-HPV	Lymph node metastases in 30% of the patients; metastases to bone and brain; good response to check-point inhibitors in one case, not confirmed in others

Abbreviations. SCC: squamous cell carcinoma; ISP: inverted sinonasal papilloma; OSP: oncocytic sinonasal papilloma; ND: not determined; HPV: human papilloma virus; HR: high risk.

**Table 2:** Molecular subgroups of sinonasal undifferentiated carcinomas.

Molecular Subgroup	Defining Genetic Findings	Histologic Subtype/Features	Immunohistochemical Markers	Other Genetic Findings	Clinical Significance
*IDH2* mutant	R172X mutations	SNUC; rarely: large cell neuroendocrine carcinoma; high grade non ITAC	Cytokeratins (simple epithelia); positive with anti-IDH2 mutant R132/R172	Distinctive hypermethylation pattern; increase in repressive trimethylation of *H3K27*; gains on chromosome arm 1q	Better DFS; specific IDH-guided therapies
*IDH2* wild- type	Absence of *IDH2* mutations	SCNEC; poorly differentiated carcinomas with NE differentiation	Variable	Frequent *ARID1A* mutations; *TP53* mutations in SCNEC; alterations in Wnt pathway genes	ND
SMARCB1 deficient	Homozygous deletion, hemizygous deletion, or truncating mutations of *SMARCB1*	Undifferentiated carcinoma with basaloid features or less frequently rhabdoid cells; high grade non ITAC	Loss of INI1; CK5/6, P63, CDX2 + in 50–60%; focal positivity for neuroendocrine markers in some cases	Loss of *NF2* and *CHEK2*, chromosome 7 gain, *TP53 V157F, CDKN2A W110*, and *CTNNB1* S45F mutations	Poorer DFS; possible treatment with EZH2 inhibitors
SMARCA4 deficient	*SMARCA4* inactivating mutations	Undifferentiated carcinoma with large or less frequently basaloid cells, sometimes rhabdoid; teratocarcinosarcoma	Loss of SMARCA4 (BRG1); limited neuroendocrine markers in many cases	Activating p.S45F mutation of β-catenin in teratocarcinosarcoma	possible treatment with EZH2 inhibitors
NUT carcinoma	*NUTM1* gene rearrangement	Uniform neoplastic population of round/polygonal cells; abrupt keratinization in 43%	Homogeneous NUT nuclear positivity; cytokeratins, p63+/−, CD34−/+	ND	Possible treatment with bromodomain inhibitors

Abbreviations: SNUC: sinonasal undifferentiated carcinoma; SCNEC: small cell neuroendocrine carcinoma; DFS: disease free survival; ND: not determined.
